# Factors influencing patients’ adherence to malaria artemisinin-based combination therapy in Kamuli District, Uganda

**DOI:** 10.1186/s12936-023-04824-8

**Published:** 2024-01-02

**Authors:** Charles Bawate, Sylvia T. Callender-Carter, Bernard Guyah, Collins Ouma

**Affiliations:** 1 Kamuli General Hospital–Kamuli District Local Government, Kamuli, Uganda; 2 Bugema University, Kampala, Uganda; 3 School of Public Health and Community Development, Maseno University, Maseno, Kenya

**Keywords:** Patients’ adherence, Artemisinin-based combination therapy, Uganda

## Abstract

**Background:**

Patients’ adherence to artemisinin-based combination therapy (ACT) is a malaria control strategy. Studies report varied experiences regarding patients’ adherence to ACT. The study aimed at determining factors influencing patients’ adherence to ACT for malaria in Kamuli, Uganda.

**Methods:**

In a longitudinal study, 1266 participants at 8 public health facilities were enrolled. Equal numbers (422) were assigned to the three arms (no follow-up, day 2 and day 4). To establish the mean difference between groups, Student t-test was used and a chi-square test was used for proportionality. A multivariate logistic regression analysis was used to establish the influence of predictor variables on the dependent variable. Statistical significance was established at *p* < 0.05.

**Results:**

A total of 844 patients were analysed. The median age was 20 years, majority (64.3%) were females. Overall patients’ adherence was 588/844 (69.7%). At bivariate level, age (t-test = 2.258, *p* = 0.024), household head (χ^2^ = 14.484, *p* = 0.002), employment status (χ^2^ = 35.886, *p* < 0.0001), patients’ preference of ACT to other anti-malarials (χ^2^ = 15.981, *p* < 0.0001), giving a patient/caregiver instructions on how to take the medication (χ^2^ = 7.134, *p* = 0.011), being satisfied with getting ACT at facility (χ^2^ = 48.261, *p* < 0.0001), patient/caregiver knowing the drug prescribed (χ^2^ = 5.483, *p* = 0.019), patient history of saving ACT medicines (χ^2^ = 39.242, *p* < 0.0001), and patient ever shared ACT medicines (χ^2^ = 30.893, *p* < 0.0001) were all associated with patients’ adherence to ACT. Multivariate analysis demonstrated that adhering to ACT is 3.063 times higher for someone satisfied with getting ACT at the facility (OR = 3.063; *p* < 0.0001), 4.088 times for someone with history of saving ACT medicines (OR = 4.088; *p* < 0.0001), 2.134 times for someone who shared ACT (OR = 2.134; *p* = 0.03), and 2.817 times for someone with a household head (OR = 2.817; *p* = 0.008).

**Conclusion:**

Patients’ adherence to ACT is generally good in the studied population. However, patients’ tendencies to save ACT for future use and sharing among family members is a threat, amidst the benefits associated with adherence. There is a need to educate all about adherence to medicines as prescribed, and tighten government medicine supply chain to avoid stock-outs.

**Supplementary Information:**

The online version contains supplementary material available at 10.1186/s12936-023-04824-8.

## Background

Globally, there were 241 million cases of malaria in 2020, an increase from 227 million in 2019 with the World Health Organization (WHO) African Region accounting for most of the increase, possibly due to the corona virus (COVID-19) pandemic [1]. The majority, 228 (95%), of malaria cases occurred in sub-Saharan Africa (SSA) [1]. The WHO surveys conducted in SSA between 2013 and 2015 revealed that a considerable proportion (36%) of febrile children did not access healthcare services. Yet among those who accessed, more patients were seen in the public sector (median 42%) than in the formal private sector (10%), and 3% in the informal private sector [2]. Also, an estimated total of US$ 9.4 billion in funding was spent on direct budgetary requirements in the eleven High Burden to High Impact Approach countries (Uganda inclusive), of which 82% came from international sources [2]. Malaria economic costs to “ill-funded” health systems and poor households are extremely high and unsustainable. More so, worker’s output is reduced [3], and school children miss school [4] for long periods due to repeated episodes of the disease. If nothing is done in a timely manner with the recent information of the emerging partial resistance to artemisinin in the WHO African region, the earlier gains made in the fight against malaria in this region could be lost [1]. Early diagnosis and prompt treatment of malaria cases remains one of the greatest strategies of managing and interrupting malaria spread [2, 5]. The patient plays an enormous role in both approaches. Patient’s ability to increase malaria knowledge and appropriate use of their prescribed anti-malarials, can reduce infections, save lives, and improve quality of life. Without concerted efforts, the WHO strategy of universal access to malaria prevention, diagnosis and treatment and the attainment of malaria-free status according to the Global Technical Strategy looks to be beyond what Uganda can achieve [6]. The success of artemisinin-based combination therapy (ACT) relies heavily on the correct patient dosage and timing of the intake of the drug [7, 8]. Several scholars have described adherent patients as those who have reported having completed the treatment course as prescribed and recommended by the healthcare provider (regarding timing and dosage) without any tablet remaining [9, 10]. Low adherence to treatment with artemisinin-based combinations has great consequences as patients who do not correctly complete their prescribed treatment are most likely to have poor recovery and exposure to early development of drug resistance [11, 12]. Non-adherence to ACT has been attributed to several factors, such as cost of ACT, forgetting to take the medicine, medication dislike [13, 14], improvement in patient’s condition and discontinued medication [15, 16], and forgetfulness, failure to understand instructions, absence of fever, vomiting of the drug by the child and caregivers’ perception that the child’s illness was not severe [17]. A systematic review of patients adherence to anti-malarial prescriptions showed variations in adherence to anti-malarials, with many of the studies showing very high adherence (90–100%) and others reporting low adherence below 50% [18]. Several studies have explored patients’ adherence to ACT across Africa, slightly above 80% adherence to ACT in SSA countries [19], with adherence levels of 29.4% in Kenya [20] to 100% in Malawi [21]. A recent study by Afaya and others posited a 47% patient adherence to ACT in Ghana [22].

According to the 2021 WHO malaria report, Uganda is among the six countries with the highest malaria deaths burden, contributing 5% of the just over half of all malaria deaths globally in 2020 [1]. A 2018–19 Ugandan malaria indicator survey report showed that malaria was more prevalent in the rural areas (such as Kamuli District) than the urban areas of the country, and Busoga region was the third most malaria affected area after West Nile and Karamoja regions, respectively [23]. Previous studies in rural Uganda districts of Kamuli, Kaliro, Pallisa and Budaka Districts and Kamuli District report a high demand for ACT both in the public and private district health facilities [24, 25]. Low patient adherence to ACT treatment is a big public health problem in Uganda in general and in Kamuli District in particular [24, 26].

Though several studies [22, 27–33] have been done elsewhere on patient adherence to ACT and associated factors to non-adherence [9, 10, 12, 14, 16, 17, 20, 32–34], there is limited knowledge in most low and rural settings of Uganda on the contribution of patient-related factors in improving their adherence to artemisinin-based combination treatment. Patient education on proper use of medicines has great potential to enhance their compliance and proper use of medicines [11, 35–37]. Therefore, this study was conducted to determine the factors influencing patients’ adherence to ACT in Kamuli district, Uganda and explore reasons for patients’ adherence or non-adherence to malaria prescriptions.

## Methods

### Design

A longitudinal design was used to determine levels of patients’ adherence to ACT and the factors influencing their adherence to ACT. First, patients were identified and enrolled into the study at eight public health facilities on exit in the months of March and April 2023. These were assigned into any of the three arms (no follow-up, follow-up on day 2 and/or 4). The no follow-up removed uncertainty about the intent to follow-up, day two follow-up helped to assess whether patients completed treatment course too early, and day 4 assessed patients delay or discontinuation of ACT. Final follow-up was on day 2 and day 4 arms only. Secondly, a research assistant visited the household on day 2 and/or day 4 to evaluate the patients’ adherence and related factors influencing their adherence to malaria treatment. Pre-tested research tools were used, and Cronbach alpha coefficient was applied to ensure internal consistency of the research tools.

### Study area

The study was conducted in Kamuli District, formerly Bugabula District in South-Eastern Uganda. The district is rural and malaria transmission occurs throughout with a mean prevalence of 24.2% (Additional file 1: Fig. S1) climaxing after March/April and August/November rains. Data was collected in the months of March and April 2023. The study focused on public health facilities as they manage more patients compared to private facilities [2, 38], and from health facility level III and above in line with the national implementation of the test and treat policy [39]. The district is served by one public hospital, two Health Centre (HC) IV’s, and 12 HC III’s.

### Study population and eligibility criteria

The study population was patients diagnosed with malaria at outpatient departments from public healthcare facilities. All age-groups with uncomplicated malaria, without any features suggestive of severe malaria [40] and had been managed according to the updated malaria treatment guidelines (test and treat policy) [6, 40] were eligible to participate. All patients with severe malaria, those with symptoms managed but not according to the current treatment guidelines and patients with co-morbidities and pregnant/breast feeding mothers were not eligible for enrollment into the study.

### Sample size

The study was powered to determine the factors influencing patients’ adherence to malaria ACT treatment in Kamuli District, Uganda. The quantitative sample size was calculated based on the formula n = Z^2^ * P(1 − P)/e^2^ [41], assuming a 95% CI, allowable error of 5% and a 46.5% patients’ adherence level to ACT [22]. The final sample size was calculated as follows: n = (1.96^2^ * 0.465 * 0.535)/0.05^2^, n = 383. Further, a loss to follow up of 10% and design effect of three arms was considered (n = 10% * 383 * 3, n = 1266 participants). However, the no follow-up arm was not studied as it masked the intention to follow-up, and these were removed from the final analysis and hence the final minimum sample size was set at n = 844.

### Sampling procedures

Eight public health facilities (one government hospital, one HC IV and six HC IIIs) in Kamuli District, Uganda were studied. The names of all HC IVs were written on small pieces of paper, folded, and put in an empty box. One facility was drawn out without replacement by simple random sampling. The same process was repeated at HC IIIs and six facilities were drawn out of the possible 12 using a simple random sampling method. As higher level facilities serve more patients [39], 300:246:120 participants were studied at hospital: HC IV:HC III, respectively.

At facility level, patients were enrolled into the study at exit to minimize the prescribers’ change in behaviour (Hawthorne effect). At HC III facilities’, about 19 people are diagnosed with malaria at OPD on each day, hence 19/3 approximates to six participants enrolled into the study per day. At HC IV, approximately 30 people are diagnosed with clinical malaria on each day resulting into 10 participants enrolled into the study per day, and at hospital at least 40 people are diagnosed with uncomplicated malaria thus, 13 participants enrolled into the study per day. A first case was identified at random and every third person who met the criteria was enrolled into the study thus systematic random sampling. In case one declined, the immediate next person would be approached. Participants were enrolled from Monday to Wednesday of every week to allow those enrolled-on Wednesday to be followed up to Saturday. Residence particulars and telephone contacts were collected to facilitate the follow-up activity in the field. Only those assigned to day 2 and 4 arms were followed up to ascertain their adherence level to treatment by recall and tablet counting and establish factors influencing their adherence to ACT prescriptions.

A pseudo-random number generator in STATA version 11 (STATA Corporation, College Station, Texas) was used in the creation of a list to assign follow-up assignments to each participant based on the enrollment order. Both the research assistants participating in enrolment and the participants themselves were blinded to the eventual follow-up assignment. The final assignment was either to no follow-up, follow-up on day 2 and follow-up on day 4.

### Data collection procedures

Eight trained research assistants led by the principal investigator (PI) constituted the data collection team. The research assistants had at least a certificate in a medical course, with 1–2 years’ working experience in the community. These were trained for 1 week in good research conduct and data collection using the various research tools by the PI. The PI provided oversight for the team and was responsible for introducing the study to the district authorities and facility in-charge and obtaining authorization to proceed with data collection. Once permission was granted, data collection in the district was commenced. As patients exited the facility, they were approached by the study staff for participation and screened for eligibility criteria. Upon provision of facility-based consent, they were enrolled into the study. The enrollment forms were forwarded to the PI who assigned the participants into any of the three arms using computer-generated numbers. A study staff then followed up the participant at his/her household on day 2 or day 4. Upon provision of a home-based consent, the research assistants collected data from consented participants using a structured questionnaire capturing information on socio-demographics, knowledge, presenting complaints, diagnosis, and treatment, socio-economic, socio-cultural and health system factors, expectations, and patients’ adherence to ACT malaria prescriptions. The field staff also sought for the blister packs for observation and counting any tablets remaining in the pack if any. The PI checked all data at the end of the day for completeness. Primary data was elucidated from the field using questionnaire, interview, and observation methods.

### Ethical considerations

Before study implementation, ethical approval was issued by the Maseno University Scientific and Ethics Review Committee (MUSERC/01122/22), the Mengo Hospital Research Ethics Committee (MH/REC/144/10-2022) and the Uganda National Council for Science and Technology (HS2576ES). Kamuli District Local Government (Office of the District Health Officer) and Kamuli Municipal Council provided local clearances. The study objectives, its benefits and potential risks and its procedures were explained to the participants and parents/guardians of the potential study children. Children were only included in the study if their parents or guardians provided written informed consent. If the participant, parent, or guardian could not read or write, a witness chosen by the participant/parent/guardian co-signed the consent/assent form.

### Data management and analysis

Data capture screens were designed in Epi-data version 3.1 with inbuilt checks and double entry command to minimize data entry errors. Data were entered and secured on a protected computer. Data were transferred from Epi-data to IBM SPSS version 20 software for statistical analyses. Exploratory analyses were done to check for cleanliness and any outliers or erroneous looking data were cross-checked and cleaned where errors were identified. Further analysis was done on tablet count response as a measure of patients’ adherence to ACT prescriptions. To establish the mean difference between groups, a student t-test was used while a chi-square test was used for proportionality. A multivariate logistic regression analysis was used to establish the influence of predictor variables on the dependent variable. Statistical significance was established at *p* < 0.05.

## Results

### Baseline characteristics of study participants and health facilities

A total of 1266 study participants were enrolled, with 422 participants in no follow-up arm not followed up and the remaining 844 participants followed up (equal numbers of 422 followed up on days 2 and 4). A total of 200 participants were followed up at Kamuli Hospital, 164 participants at Namwendwa HC IV, and 80 participants at each of six remaining HC IIIs. At each of the facilities, half of the participants were followed up on either day 2 or day 4. Majority 543 (64.3%) of participants were females. The median age of all patients was 20 (IQR 9.25, 32) years, with an overall enrollment of 481 (57%) aged above 18 years. The majority, 517 (61.3%) of participants were not married, and this was consistent across all facilities except at Nabirumba HC III where only half (50%) were not in a relationship. The overall mean size of the households was 6.09 (minimum = 1, maximum = 16) members, with highest mean size (mean = 8.04, minimum = 2, maximum = 13) at Balawoli HC III. Overall, the majority, 666 (79%) of households were led by fathers, and least 19 (2.3) by siblings. Generally, the majority of 261 (30.9%) participants were daughters followed by 218 (25.8%) partners, and 4 (0.5%) grandparents by relationship to household head. The majority, 683 (80.9%) of households had a mobile telephone set, with all (100%)—Namwendwa HC IV, least 33 (41.2%) households with a telephone visiting Kitayunjwa HC III (see Table 1).

**Table 1:** Baseline characteristics of study participants and health facilities

Variable	All	Kamuli hospital	Namwendwa HC IV	Balawoli HC III	Nabirumba HC III	Kitayunjwa HC III	Bupadhengo HC III	Bugulumbya HC III	Busota HC III
Patient characteristics									
Day 2 number	422	100	82	40	40	40	40	40	40
Day 4 number	422	100	82	40	40	40	40	40	40
Females, n (%)	543 (64.3)	128 (15.2)	91 (10.8)	55 (6.5)	60 (7.1)	47 (5.6)	45 (5.3)	63 (7.5)	54 (6.4)
Age in years, median (IQR)	20 (9.25, 32.0)	23 (19.0, 39.0)	17.5 (4, 35)	27 (18, 51)	21.5 (11, 28)	9 (4, 18.75)	15.5 (5.25, 23)	14 (6, 21)	23.5 (15, 42)
Age category in years									
0–18, n (%)	363 (43)	42 (21)	84 (51.2)	23 (28.7)	31 (38.8)	60 (75)	49 (61.3)	51 (63.7	23 (28.7)
19 and above, n (%)	481 (57)	158 (79)	80 (48.8)	57 (71.3)	49 (61.2)	20 (25)	31 (38.7)	29 (36.2)	57 (71.3)
Marital status									
Married, n (%)	327 (38.7)	78 (39)	74 (45.1)	38 (47.5)	40 (50)	20 (25)	15 (18.8)	23 (28.7)	39 (48.8)
Not married, n (%)	517 (61.3)	122 (61)	90 (54.9)	42 (52.5)	40 (50)	60 (75)	65 (81.2)	57 (71.2)	41 (51.2)
Household size									
Mean (min, max)	6.09 (1, 16)	4.69 (1, 10)	7.29 (3, 15)	8.04 (2, 13)	6.01 (1, 13)	6.74 (2, 16)	5.49 (1, 8)	5.64 (2, 16)	5.69 (1, 15)
Household head									
Father, n (%)	666 (79)	139 (69.5)	161 (98.2)	58 (72.5)	68 (85)	68 (85)	52 (77.5)	57 (72.2)	53 (66.2)
Mother, n (%)	100 (11.9	39 (19.5)	0 (0)	8 (10)	3 (3.8)	4 (5)	16 (20)	14 (17.7)	16 (20)
Sibling, n (%)	19 (2.3)	10 (5)	3 (1.8)	0 (0)	1 (1.2)	0 (0)	2 (2.5)	2 (2.5)	1 (1.2)
Grandparent, n (%)	58 (6.9)	12 (6)	0 (0)	14 (17.5)	8 (10)	8 (10)	0 (0)	6 (7.6)	10 (12.5)
Relationship to household head									
Son, n (%)	166 (19.7)	32 (16)	47 (28.7)	10 (12.5)	12 (15)	21 (26.2)	26 (32.5)	11 (13.8)	7 (8.8)
Daughter, n (%)	261 (30.9)	62 (31)	49 (29.9)	15 (18.8)	25 (31.2)	33 (41.2)	32 (40)	34 (42.5)	11 (13.8)
Sibling, n (%)	17 (2)	8 (4)	1 (0.6)	0 (0)	1 (1.2)	0 (0)	2 (2.5)	2 (2.5)	3 (3.8)
Grandchild, n (%)	39 (4.6)	8 (4)	0 (0)	4 (5.0)	4 (5)	8 (10)	0 (0)	6 (7.5)	9 (11.2)
Grandparent, n (%)	4 (0.5)	4 (2)	0 (0)	0 (0)	0 (0)	0 (0)	0 (0)	0 (0)	0 (0)
Self, n (%)	139 (16.5)	38 (19)	22 (13.4)	25 (31.2)	10 (12.5)	5 (6.2)	11 (13.8)	4 (5)	24 (30)
Partner, n (%)	218 (25.8)	48 (24)	45 (27.4)	26 (32.5)	28 (35)	13 (16.2)	9 (11.2)	23 (28.7)	26 (32.5)
Homestead with telephone contact									
Yes, n (%)	683 (80.9)	184 (92)	164 (100)	75 (93.8)	42 (52.5)	33 (41.2)	51 (63.7)	59 (73.8)	75 (93.8)

HC: Health Centre; n: number; %: percentage; Min–M: minimum; Max: maximum; IQR: inter quantile range

### Patients’ adherence to malaria treatment

The overall patients’ adherence to ACT by recall of tablets remaining in the blister pack was 587/843 (69.6%) and 588/844 (69.7%) by tablet counting by the research team. The majority 663/758 (87.5%) of respondents by recall had adhered to the treatment using ACT in their previous malaria episode (see Table 2).

**Table 2:** Patients’ adherence to ACT treatment

Variable	Adherence status	
	Adhered n (%)	Did not adhere, n (%)
Overall		
Recall	587 (69.6)	256 (30.4)
Tablet count	588 (69.7)	256 (30.3)
Previous adherence to treatment in the last malaria episode		
Recall	663 (87.5)	95 (12.5)

n: number; %: percentage

### Factors influencing patients’ adherence to ACT malaria prescriptions in Kamuli District, Uganda

Based on bivariate analysis, socio-demographic factors; age (T-test = 2.258, *p* = 0.024, 95% CI = 0.391–5.594) and household head (χ^2^ = 14.484, *p* = 0.002) were statistically associated with patients’ adherence to ACT, with relationship to household head (χ^2^ = 10.95, *p* = 0.09) showing borderline statistical significance (Table 3). However, gender (χ^2^ = 0.178, *p* = 0.673), marital status (χ^2^ = 2.276, *p* = 0.131) and household size (T-test = − 1.442, *p* = 0.15, 95% CI = − 0.677 to 0.104) did not have a significant association with appropriate patient taking of ACT. Employment status (χ^2^ = 35.886, *p* < 0.0001) had a significant association with patients’ adherence to ACT treatment, and source of information in a homestead (χ^2^ = 6.866, *p* = 0.076) had a borderline statistical association with patients’ adherence to ACT among the socio-economic factors. Education level (χ^2^ = 7.427, *p* = 0.191), monthly income (T-test = − 0.001, *p* = 0.999, 95% CI = − 40433.157 to 40381.786), household means of transport to health facility (χ^2^ = 1.043, *p* = 0.307), and nature of family house (χ^2^ = 5.671, *p* = 0.129) did not have a significant association with patients’ adherence to ACT prescription. Patients’ preference to ACT to other anti-malarials (χ^2^ = 15.981, *p* < 0.0001) was statistically associated with patients’ adherence to ACT, with perceived preference of drug formulation (χ^2^ = 5.875, *p* = 0.053) having a borderline significant association. Socio-cultural factors of religion (χ^2^ = 4.723, *p* = 0.451), being satisfied with waiting time to get services (χ^2^ = 1.63, *p* = 0.213), and taking herbal medicine together with ACT (χ^2^ = 0.803, *p* = 0.387) did not have any statistical association with patients’ adherence to ACT prescriptions. Giving a patient/caregiver instruction on how to take the medication (χ^2^ = 7.134, *p* = 0.011), being satisfied with getting ACT at facility (χ^2^ = 48.261, *p* < 0.0001), patient/caregiver knowing the drug prescribed (χ^2^ = 5.483, *p* = 0.019), patient history of saving ACT medicines (χ^2^ = 39.242, *p* < 0.0001), and patient ever shared ACT medicines (χ^2^ = 30.893, *p* < 0.0001) were all associated with patients’ adherence to ACT prescriptions. However, health system factors of patient taking first dose at the facility under close observation of a qualified professional (χ^2^ = 0.044, *p* = 0.824), patient being seen by a qualified professional (χ^2^ = 0.085, *p* = 0.81), use of ACT package as visual aid for instructions (χ^2^ = 1.96, *p* = 0.173), knowing the right dosage (χ^2^ = 1.26, *p* = 0.286), patient presenting with fever (χ^2^ = 0.045, *p* = 0.836), and type of malaria diagnostic method used in the diagnosis of malaria (χ^2^ = 1.122, *p* = 0.571) did not have a statistically significant association with patients’ adherence to ACT prescriptions (Table 3).

**Table 3:** Bivariate analysis of factors influencing patients’ adherence to ACT treatment in Kamuli District

Variable	Adhered	Did not adhere	Test-statistic	p-value	95% CI (lower, upper)
Socio-demographic factors					
Age	Mean Diff = 2.993		2.258	0.024*	(0.391, 5.594)
Gender					
Male	207 (66.8)	94 (31.2)	0.178	0.673	
Female	381 (70.2)	162 (29.8)			
Marital status					
In relationship	370 (71.6)	147 (28.4)	2.276	0.131	
Not in a relationship	218 (66.7)	109(33.)			
Household size	Mean Diff = − 0.287		− 1.442	0.150	(− 0.677, 0.104)
Household head					
Father	451 (67.7)	215 (32.3)	14.484	0.002*	
Mother	79 (79)	21 (21)			
Sibling	9 (47.4)	10 (52.6)			
Grandparent	48 (82.8)	10 (17.2)			
Relationship to household head					
Son	119 (71.7)	47 (28.3)	10.95	0.09**	
Daughter	178 (68.2)	83 (31.8)			
Sibling	8 (47.1)	9 (52.9)			
Grandchild	30 (76.9)	9 (23.1)			
Grand parent	3 (75)	1 (25)			
Self	107 (77)	32 (23)			
Partner	143 (65.6)	75 (34.4)			
Socio-economic factors					
Education level					
Primary	230 (73.2)	84 (26.8)	7.427	0.191	
Below certificate	174 (66.7)	87 (33.3)			
Certificate holder	123 (65.4)	65 (34.6)			
Diploma holder	13 (68.4)	6 (31.6)			
Masters/PGD/PhD	4 (100)	0 (0)			
No formal education	44 (75.9)	14 (24.1)			
Employment status					
Formal employment	52 (71.2)	21 (28.8)	35.886	0.000*	
Informal employment	217 (59.1)	150 (40.9)			
Not employed at all	319 (79)	85 (21)			
Monthly income	Mean Diff = − 25.686		− 0.001	0.999	(− 40433.157, 40381.786)
Has transport means to facility					
Yes	425 (68.7)	194 (31.3)	1.043	0.307	
No	162 (72.3)	62 (27.7)			
Source of information in homestead					
Radio	335 (66.3)	170 (33.7)	6.866	0.076**	
Television	75 (76.5)	23 (23.5)			
Both radio and television	91 (73.4)	33 (26.6)			
No	87 (74.4)	30 (25.6)			
Nature of family house					
Rented	135 (74.6)	46 (25.4)	5.671	0.129	
Owned—permanent	266 (65.8)	138 (34.2)			
Owned—semi permanent	157 (72.4)	60 (27.6)			
Owned—temporary	30 (71.4)	12 (28.6)			
Socio-cultural factors					
Religion					
Ccatholic	130 (73.9)	46 (26.1)	4.723	0.451	
Anglican	267 (69.7)	116 (30.3)			
Muslim	82 (67.2)	40 (32.8)			
Born again Christian	105 (68.2)	49 (31.8)			
Traditional believer	1 (50)	1 (50)			
Seventh Day Adventist	3 (42.9)	4 (57.1)			
Prefer ACT to other antimalarials					
Yes	477 (73)	176 (27)	15.981	< 0.0001*	
No	110 (57.9)	80 (42.1)			
Perceived preference of drug formulation					
Tablet	488 (70.8)	201 (29.2)	5.875	0.053**	
Syrup	18 (52.9)	16 (47.1)			
Injection	51 (64.6)	28 (35.4)			
Satisfied with waiting time to get service					
Yes	370 (71.3)	149 (28.7)	1.63	0.213	
No	210 (67.1)	103 (32.9)			
Took herbal medicine with ACT					
Yes	143 (67.5)	69 (32.5)	0.803	0.387	
No	442 (70.7)	183 (29.3)			
Health system factors					
Patient/caregiver given instructions on how to take the medicine					
Yes	542 (71.1)	220 (28.9)	7.134	0.011*	
No	46 (56.8)	35 (43.2)			
Patient took first dose of ACT at the facility under observation of health worker					
Yes	75 (68.8)	34 (31.2)	0.044	0.824	
No	513 (69.8)	222 (30.2)			
A qualified health worker saw patient					
Yes	524 (70)	225 (30)	0.085	0.81	
No	63 (68.5)	29 (31.5)			
ACT package used as visual aid for instructions					
Yes	323 (67.9)	153 (32.1)	1.960	0.173	
No	264 (72.3)	101 (27.7)			
Know right dosage of ACT					
Yes	508 (70.4)	214 (29.6)	1.260	0.286	
No	79 (65.3)	42 (34.7)			
Satisfied with getting ACT medicine at facility					
Yes	515 (74.9)	173 (25.1)	48.261	< 0.0001*	
No	72 (46.5)	83 (53.5)			
Patient present with fever					
Yes	569 (69.7)	247 (30.3)	0.045	0.836	
No	19 (67.9)	9 (32.1)			
Malaria diagnostic method					
Microscopy	308 (70)	132 (30)	1.122	0.571	
RDT	277 (69.6)	121 (47.3)			
Both	3 (50)	3 (50)			
Patients know the ACT prescribed					
Yes	465 (67.9)	220 (32.1)	5.483	0.019*	
No	123 (77.4)	36 (22.6)			
Participant ever saved ACT medicines (history of saving ACT)					
Yes	77 (51.7)	72 (48.3)	39.242	< 0.0001*	
No	178 (25.7)	515 (74.3)			
Participant ever shared ACT medicines					
Yes	67 (50.8)	65 (49.2)	30.893	< 0.0001*	
No	189 (26.5)	523 (73.5)			

*ACT* artemisinin-based combination therapy, *RDT* malaria rapid diagnostic test, *Mean Diff* mean difference, *CI* confidence interval

*Statistically significant

**Borderline statistical significance

At multilevel logistic regression analysis, the odds of one adhering to ACT prescription is 3.063 times higher for someone who reports being satisfied with getting ACT at the facility (OR = 3.063; *p* < 0.0001; 95% CI = 1.857–5.051), 4.088 times for someone with history of saving ACT medicines (OR = 4.088; *p* < 0.0001; 95% CI = 2.165–7.721), 2.134 times for someone who ever shared ACT (OR = 2.134; *p* = 0.03; 95% CI = 1.078–4.224), and 2.817 times for someone with a household head (OR = 2.817; *p* = 0.008; 95% CI = 1.306–6.077), and all these factors were statistically significant (Table 4).

**Table 4:** Logistic regression predicting patients’ adherence to ACT malaria prescriptions in Kamuli District, Uganda

Variable	Sig.	OR	95% CI	
			Lower	Upper
Age of participant	0.652	1.004	0.986	1.024
Marital status (in relationship)	0.637	0.825	0.371	1.834
Household head (father)	0.008			
Household head (mother)	0.008	2.817	1.306	6.077
Household head (sibling)	0.289	0.336	0.045	2.527
Household head (grandparent)	0.048	14.730	1.020	212.672
Relationship to head of family	0.601			
Relationship to family head (daughter)	0.622	0.872	0.504	1.506
Relationship to family head (sibling)	0.962	0.948	0.107	8.425
Relationship to family head (grandchild)	0.042	0.053	0.003	0.897
Relationship to family head (grandparent)	0.538	0.374	0.016	8.524
Relationship to family head (self)	0.859	1.091	0.415	2.870
Relationship to family head (partner)	0.974	1.016	0.398	2.595
Education level (no formal education)	0.543			
Education level (primary)	0.086	2.289	0.889	5.891
Education level (below certificate)	0.152	2.129	0.757	5.982
Education level (certificate holder)	0.062	2.785	0.950	8.170
Education level (diploma)	0.244	2.616	0.518	13.207
Education level (Masters/PGD/PhD)	0.999	2477991249.571	0.000	
Employment status (not employed)	0.060			
Employment status (formal)	0.254	0.625	0.279	1.402
Employment status (informal)	0.018	0.534	0.318	0.897
Monthly income	0.524	1.000	1.000	1.000
Source of information (Nothing)	0.302			
Source of information (radio)	0.513	1.251	0.640	2.443
Source of information (TV)	0.151	1.937	0.786	4.773
Source of information (TV and Radio)	0.133	1.943	0.817	4.621
Nature of family house (rented)	0.089			
Nature of family house (permanent)	0.091	0.629	0.368	1.076
Nature of family house (semi-permanent)	0.775	1.091	0.600	1.984
Nature of family house (temporary)	0.712	1.208	0.443	3.297
Prefer ACT to other antimalarials	0.053	1.626	0.995	2.657
Preferred drug formulation (tablet)	0.466			
Preferred drug formulation (syrup)	0.216	0.581	0.246	1.374
Preferred drug formulation (injection)	0.873	0.948	0.495	1.815
Given instructions (yes)	0.257	1.524	0.736	3.158
Package used as a visual aid (yes)	0.770	1.071	0.676	1.699
Know right dosage (yes)	0.054	0.536	0.284	1.012
Getting ACT at facility(yes)	0.000	3.063	1.857	5.051
Ever saved ACT (history of saving ACT) (yes)	0.000	4.088	2.165	7.721
Ever shared ACT (yes)	0.030	2.134	1.078	4.224
Constant	0.001	0.065		

*OR* odds ratio, *CI* confidence interval, *TV* television

sig. p-value

### Reasons for not completing, benefits of completing, reasons for saving and sharing, side effects and dangers of not completing prescribed antimalarial treatment

The main reasons for not completing prescribed ACT were not having money to buy drugs 70/208 (33.7%), delay in buying medicines 39/208 (18.8%), forgetfulness 31/208 (14.9%), and not having money to buy full dose 26/208 (12.5%). The main benefits of adhering to prescription were complete cure and feel good after taking the medicine 637/779 (81.8%) and reduction in malaria spread 79/779 (10.1%). The main reasons for saving ACT were for future use 74/110 (74%) and felt better and stopped taking 31/110 (28.2%). The main reasons for sharing ACT were ACT is expensive and no money to buy always 67/172 (38.9%), shared with sick child 57/172 (31.4%) and shared with sick family member 31/172 (18%). The major side effects experienced by patients were vomiting 187/404 (46.3%), dizziness 72/404 (17.8%), general body weakness 53/404 (13.1%), and bad smell 34/404 (8.4%). The dangers of not completing ACT according to prescription were no complete cure and disease continue 263/657 (40%), death 154/657 (23.5%), disease progresses and becomes severe 90/657 (13.7%), drug resistance 65/657 (9.9%), and results in more malaria spread 47/657 (7.2%) (Additional file 2).

## Discussion

This study determined the factors influencing patients’ adherence to malaria ACT treatment. Final analysis on adherence in this study was measured by tablet counting and patient recall, particularly with dosing and timing. Patients’ adherence to ACT was just above average at 69.7%. The current study’s finding is consistent with a Kenyan study seeking services from public health facilities [42]. However, it contradicts those reporting low adherence to ACT below 50% [18, 20, 22, 34] and those reporting high adherence above 80% [17, 19, 21, 33]. The variations in the current study finding versus other studies might be due to varied methodologies employed per study [33, 34]. In addition, the previous studies were conducted during the era of chloroquine and primaquine as drugs in the management of malaria. Yet, in the Ugandan study in which an integrated approach was used, the high adherence to ACT could have been masked by the effect of poly-pharmacy [17]. Additional findings in the current study shows that the contribution of drug stock out and failure for patients to buy drugs was linked to the adherence to ACT. The current study also demonstrated age to be statistically associated with patients’ adherence to ACT. In addition, adherence was further shown to be higher among the youngest age which was consistent with a Ghanian study [27]. However, the current findings contradict the findings in previous studies [16, 18–20]. These differences in the current versus previous studies could be due to extra care parents/caregivers give when attending to the young patients in the current study population, which then enhances adherence levels in this category. However, the adults themselves are always trapped with other engagements and forgetfulness without anyone reminding them, thus leading to low adherences. The study also revealed that household head had a statistical association with patients’ adherence to ACT, and those with a household head were 2.817 times more likely to adhere to ACT prescriptions than those without a household head. This finding shows the role of figure-head in a family in an African setting when it comes to accessing, utilization, and decision-making in regards to healthcare services. The study revealed that relationship to household head, gender, marital status, and household size were not statistically associated with patients’ adherence to ACT. These factors were not associated with adherence to ACT since there is no discrimination among family members based on above constructs in traditional African settings and every member of the family is treated with due respect especially when it comes to health-seeking behaviour to malaria. Thus, the figurehead ensures the health of the family members at all times.

There was also a significant association of patients’ employment status with ACT adherence, with those employed formally 1.402 times more likely to adhere than those not employed at all. This is in line with studies which have shown that families spend a lot in accessing healthcare services [43, 44]. Therefore, only those who are engaged in some income generating activity can have access to money to spend on their healthcare, more so, in times of scarcity of drugs at these public facilities. Other observation was that patients’ preference of ACT to other antimalarials had a statistical significance with adherence. This contradicts a Kenyan study that showed a statistical association between patient’s dislike of ACT and adherence [14]. The behaviour could be attributed to the perceived knowledge about the efficacy of the drug. The study also revealed that source of health information, education level, monthly income, household means of transport to health facility, perceived preference of drug formulation, and nature of family house were not statistically associated with patients’ adherence to ACT. The current study finding contradicts other studies which found a statistical association between education and patient adherence to ACT [14, 30, 45]. Moreover, an Ethiopian study had shown earlier that illiteracy is a major hindering factor of patient adherence to ACT [29]. It is believed that proper education empowers and enables one to make informed decisions and discern between what is wrong and right about one’s health. Therefore, there is need to study the value of a tailored community education on malaria and use of ACT to provide evidence on what really works. The current study findings are consistent with another Ugandan study by Niringiye and Douglason showing no association between income and patients’ adherence to ACT [46]. This could be due to poor health-seeking and utilization behaviours of the society. However, further studies are needed to explore this trend.

There was also significant association of giving a patient/caregiver instruction on how to take the medication with adherence. This is in line with a study that showed that giving clear instructions to patients and caregivers on how to correctly take the medicines is key in their adherence to treatment [37]. More so, Kalyango and others noted that failure to understand instructions results in non-adherence [17]. Clear instructions can improve knowledge and demystify several factors and improve adherence to medication. The more the patient has correct information about the treatment, the better the adherence to the guidelines provided hence adherence to treatment.

There was a significant relationship between being satisfied with getting ACT at facility and adherence, and those who got ACT medicines at facilities were 3.063 times more likely to adhere to ACT prescriptions than those who did not get it. It has been noted that public health facilities experience stock-outs of ACT medicines and diagnostic services for malaria [47], and artemisinin-based combinations are costly at the private market [48], hence those who get it for free are always satisfied with the service and hence can improve their adherence to treatment. In addition, there was a significant association between patients who had history of saving ACT medicines and adherence, and those who had history of saving ACT were 4.088 times more likely to adhere to ACT prescriptions. This could be explained by the frequent stock-out of ACT medicines [47] and heightened demand [24, 25] at the public facilities and thus it is expected that patients would prefer to save for future use and feeling better before dosage completion. It is further hypothesized that community members tend to seek services and forge sickness to obtain drugs at public facilities with hope of using them during times of scarcity. However, this was beyond the scope of this study and needs to be explored more.

The study also revealed a statistical association between patients who shared ACT medicines and adherence, and those who shared were 2.134 times more likely to adhere. As revealed in this study, those who shared medicine did so due to excessive cost of the drug and could share with a sick family member. Though the current study shows its power to increase their adherence to ACT prescription, it could easily downplay and expose them to suboptimal doses with its consequences [11, 12]. Further, the behaviour of using drugs without prior testing during this era of test and treat [39] is very dangerous and counterproductive to government efforts of fighting this old enemy. The study further showed that religion, satisfaction with waiting time to get services, taking herbal medicines together with ACT, taking first dose of ACT at facility under observation of qualified professional, patient being seen by qualified personnel, use of ACT package as a visual aid for instructions, patient knowledge of right dosage, history of fever and type of malaria diagnostic technique did not have a statistical association with patients’ adherence to ACT prescriptions. As in a study by Afaya et al. [22, 49], Christians dominated the study and healthcare workers should operate in harmony with clients and relatives in decision making by seeking their opinions and beliefs to avoid conflict with their underlying religious ethos. Unlike in a Kenyan study [14], waiting time did not contribute significantly to patient adherence. The difference could be in the time measurements employed and the use of RDTs which are more rapid and fast [50] in the current study. Also on the use of herbal or co-current taking of herbal medicine, the current study did not establish a similar trend as in the study by Yakasai et al. [37], where patients preferred to use of herbals due to high costs involved in disease management at health facilities. This could be due to some ongoing health education talks and sensitization in this community. Knowledge of right dosage of ACT was not associated with patients’ adherence in the current study, contrary to previous studies [14, 30, 45, 51]. This could be due to differences in the study areas and designs employed. The current study revealed that the vast knowledge of benefits arising out of good adherence to treatment does not really result in improved adherence to ACT prescriptions. Therefore, knowledge alone is not enough to improve adherence. Other factors like availability of drugs (Tables 3 and 4) play a role in influencing patients’ adherence to ACT prescriptions. The use of ACT package as a visual aid for instructions was just average despite its power to enhance one’s adherence through boost of memory. Similar findings were posited in another Ugandan study [35], however, a Malawian study showed statistical association [32]. This could have affected the adherence of patients in this study. It has also been revealed [35], just like in in the current study that history of fever and quick recovery from symptoms results in non-adherence of patients. Taking of first dose at facilities was very low and not statistically associated with patients’ adherence unlike in a Malawian study [32]. This is majorly attributed to lack of safe drinking water supplies at facilities/dispensing points. Further, there is no strict law requiring malaria directly observed treatment. Further still, the study revealed that patients are more concerned with receiving a service in-terms of consultation, diagnosis and drug(s) and not knowing which healthcare worker offered it. This is compounded by many public health workers wearing uniforms without proper identification, for example name and cadre.

Non-adherence to ACT in this study has been attributed to several factors such as lack of money to buy medicines, delay in buying the medicines in line with previous studies [13, 14], feeling better and stopping to take medicine [15, 16], forgetfulness, vomiting of drugs [17]. Patients’ visiting public facilities expect to find all medicines at all times and failure to get them results in delays in making decisions to buy and sometimes fail to buy as they expect them for free and sometimes lack money. Therefore, the MoH should provide sufficient medicines including anti-malarials to all public facilities. Other factors were saving for future use, loss of drugs, sharing of drugs, unpleasant smell of drugs, taking of herbs instead of ACT, all of which are modifiable by tailored education. During the study time, some facilities were experiencing ACT stock-out. As the National Warehouse was preparing to deliver, the community remained generally poor, and ACT is expensive on the open market. Further, the current study shows that non-adherence is a recipe for poor disease prognosis and development of severe disease, lack of complete cure, death, disease recurrence, drug resistance and more disease spread as has been noted in other studies [11, 12]. To avert the current situation and to reap the full benefits of proper adherence, patients’ education on proper usage of ACT in-terms of dosage and timing, its health benefits and related adverse events should aggressively be conducted by professionals as previously noted [52], and this could improve their overall adherence to prescription and health guidelines.

## Limitations

The study was conducted only in public health facilities, which then limits generalizability to only public facilities. Secondly, it was conducted in one district with high malaria transmission, which in essence might not apply in other districts with less malaria transmissions. Health Centre II’s per policy do not have functional laboratories, hence were not studied. This limits generalization at all levels of healthcare system continuum. Measuring adherence based on pill count and recall could introduce bias as a result of subject intentional throw away of tablets, and/or forgetfulness. Such biases were minimized through careful consideration of the two methods. However, another study based on chemical analysis of drug levels in the body could be done but this was beyond the scope of the study.

## Conclusion

The study found a fairly above average level (69.7%) of patients’ adherence to ACT medication. However, patents’ knowledge of side effects, perceived benefits, and dangers of non-compliance to ACT were not enough to have all patients adhere correctly to ACT prescriptions. Absence of ACT, lack of money to buy ACT medicines, need to save for future use and medicines sharing resulted into a substantial number of patient’s not adhering to treatment. To maximize adherence to guidelines, the supply chain of ACT needs to be tightened to avoid recurrent stock-outs at public facilities. Given lack of proper knowledge on what really works and diversity in community beliefs, a community education intervention to provide guidance on best practices could result in better patients’ practices in Uganda.

### Supplementary Information

**Additional file 1: Figure S1.** Map showing malaria endemicity in Uganda.

**Additional file 2.** Table showing reasons for not completing, benefits of completing, reasons for saving and sharing, side effects and dangers of not completing ACT treatment.

## Data Availability

The datasets used and/or analysed during the current study are available from the corresponding author on a reasonable request.
