# Supplementary material for: Factors influencing patients’ adherence to malaria artemisinin-based combination therapy in Kamuli District, Uganda
Source: Malar J. 2024 Jan 2;23:1. doi: 10.1186/s12936-023-04824-8 (PMC10759708; doi:10.1186/s12936-023-04824-8)
Supplement: Supplementary file 1 — Additional file 1: Figure S1. Map showing malaria endemicity in Uganda. [file 12936_2023_4824_MOESM1_ESM.docx]

Additional file 1: Map showing Malaria endemicity in Uganda


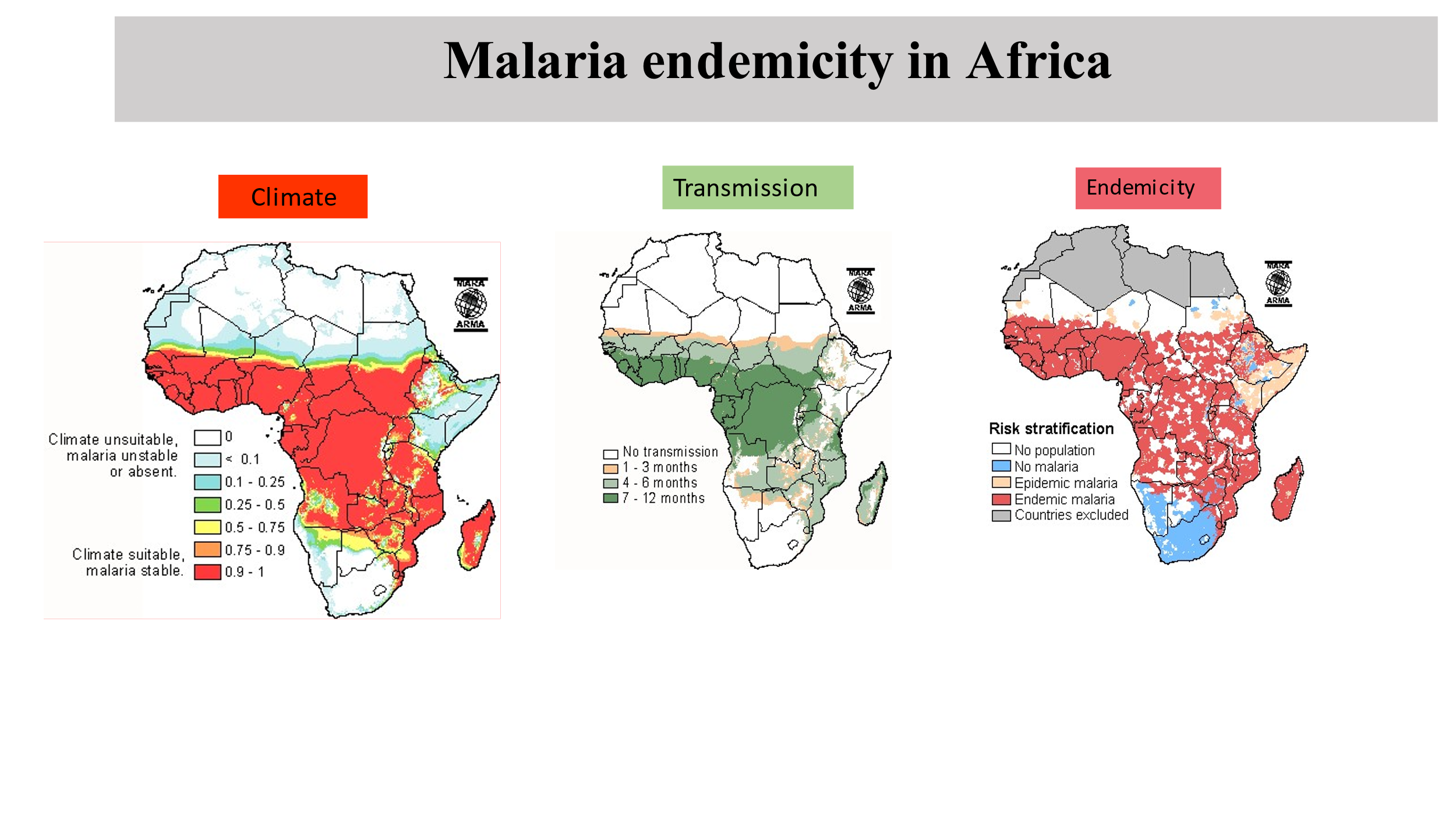


Kamuli, Uganda

Figure S1. Maps showing malaria favourable climate, transmission and endemicity Africa and Uganda
