# Supplementary material for: Factors influencing patients’ adherence to malaria artemisinin-based combination therapy in Kamuli District, Uganda
Source: Malar J. 2024 Jan 2;23:1. doi: 10.1186/s12936-023-04824-8 (PMC10759708; doi:10.1186/s12936-023-04824-8)
Supplement: Supplementary file 2 — Additional file 2. Table showing reasons for not completing, benefits of completing, reasons for saving and sharing, side effects and dangers of not completing ACT treatment. [file 12936_2023_4824_MOESM2_ESM.docx]

Additional file 2: Table showing reasons for not completing, benefits of completing, reasons for saving and sharing, side effects and dangers of not completing ACT treatment

| **Variable** | **Frequency/ N** | **Percentage / %** |
| --- | --- | --- |
| **Reasons for not completing ACT as prescribed** | | |
| Took an injection | 1 | 0.5 |
| Felt better and stopped taking medicine | 39 | 18.8 |
| Delayed buying medicines | 9 | 4.3 |
| Saved some for future use | 3 | 1.4 |
| Drugs fell before taking them | 4 | 1.9 |
| Forgetfulness | 31 | 14.9 |
| Shared medicines | 9 | 4.3 |
| Did not have money to buy full dose | 26 | 12.5 |
| Did not have money to buy drugs | 70 | 33.7 |
| Allergic to ACT | 1 | 0.5 |
| Overtook medicines | 5 | 2.4 |
| Was not improving and stopped | 1 | 0.5 |
| Drugs smell badly and had to stop taking | 4 | 1.9 |
| Vomited drugs | 3 | 1.4 |
| Took herbal instead of ACT | 2 | 1 |
| **Benefits of completing ACT treatment** | | |
| Complete cure and feel good | 637 | 81.8 |
| Prevents drug resistance | 14 | 1.8 |
| Prevents death from malaria | 7 | 0.9 |
| Reduces disease progression to severe state | 34 | 4.4 |
| Reduces on malaria spread | 79 | 10.1 |
| Helps one to take time without falling sick again | 4 | 0.5 |
| Don't know | 4 | 0.5 |
| **Reasons for saving Artemisinin-based combination treatment** | | |
| Felt better and stopped taking | 31 | 28.2 |
| Got tired of taking | 1 | 0.9 |
| Not aware of need to complete after feeling well | 1 | 0.9 |
| Future use | 74 | 67.3 |
| Forgetfulness | 2 | 1.8 |
| Used herbs and saved ACT | 1 | 0.9 |
| **Reasons for sharing Artemisinin-based combination treatment** | | |
| ACT is expensive and no money to buy always | 67 | 38.9 |
| Shared with sick child | 54 | 31.4 |
| Shared with sick family member | 31 | 18 |
| Long waiting time at public facility | 1 | 0.6 |
| Had remainder drugs previous episode | 5 | 2.9 |
| Calm high fever before reaching facility | 2 | 1.2 |
| Felt better and shared with other the rest | 5 | 2.9 |
| Did not want to go back to facility | 7 | 4.1 |
| **Side effects of ACT** | | |
| Drug reactions | 1 | 0.3 |
| Unpleasant smell | 34 | 8.4 |
| General Body weakness | 53 | 13.1 |
| Stomach-ache | 1 | 0.3 |
| Loss of appetite | 3 | 0.7 |
| Nausea | 19 | 4.7 |
| Vomiting | 187 | 46.3 |
| Diarrhoea | 11 | 2.7 |
| Joint pains | 1 | 0.3 |
| Constipation | 5 | 1.2 |
| Dizziness | 72 | 17.8 |
| Sweating | 5 | 1.2 |
| Headache | 7 | 1.7 |
| Dry mouth | 3 | 0.7 |
| Drowsiness | 1 | 0.3 |
| Sleeplessness | 1 | 0.3 |
| **Dangers of not completing Artemisinin-based combination treatment** | | |
| Disease progresses and becomes severe | 90 | 13.7 |
| Aneamia develops | 12 | 1.8 |
| Increases costs of malaria disease later | 2 | 0.3 |
| Redevelop malaria | 20 | 3 |
| Drug resistance | 65 | 9.9 |
| No complete cure and disease continue | 263 | 40 |
| Death | 154 | 23.5 |
| Develop general body weakness | 4 | 0.6 |
| Results in more malaria spread | 47 | 7.2 |

Table legend: N – frequency, % - percentage
